# Exercise intensity‐dependent cardiac telocyte expansion is associated with physiological growth despite JAK/STAT pathway inactivity in male Wistar rats

**DOI:** 10.1113/EP093390

**Published:** 2026-01-31

**Authors:** Mahboobeh Borjian Fard, Siroos Choobineh, Rahman Soori, Zohreh Mazaheri

**Affiliations:** ^1^ Department of Exercise Physiology, Faculty of Sport Sciences and Health University of Tehran Tehran Iran; ^2^ Basic medical science research center histogenotech company Tehran Iran

**Keywords:** cardiac hypertrophy, heart, high‐intensity interval training, IL‐6, JAK/STAT pathway, telocytes

## Abstract

While exercise induces physiological cardiac growth, the underlying cellular mechanisms remain incompletely understood. This study investigated the role of cardiac telocytes (TCs) and the Janus kinase (JAK)/signal transducer and activator of transcription (STAT) pathway in mediating exercise intensity‐dependent cardiac adaptation. Twenty‐four male Wistar rats were assigned to control (CTRL), high‐intensity interval training (HIIT) or low‐intensity interval training (LIIT) groups for 8 weeks. Physiological hypertrophy was assessed via heart weight/body weight ratio, left ventricular wall thickness, cardiomyocyte size and number. Cardiac TCs were quantified by immunofluorescence (CD34–platelet‐derived growth factor receptor (PDGFR)‐α/β). Gene expression of IL‐6, cardiotrophin‐1 (CTF1), GP130, JAK2, STAT3 and GATA4 was analysed by qPCR, and interleukin (IL)‐6 protein levels were measured by ELISA. Both HIIT and LIIT robustly induced physiological cardiac hypertrophy and cardiomyogenesis, with HIIT producing a significantly greater response. This was accompanied by a significant, intensity‐dependent expansion of the cardiac TC population in both HIIT and LIIT groups compared to CTRL, with HIIT inducing a greater increase than LIIT (*P* < 0.001). Furthermore, GATA4 expression, a marker of cardiac stem cell activation, was significantly upregulated in both trained groups. While cardiac IL‐6 gene expression and protein levels were elevated, particularly after HIIT (*P* = 0.003), the core components of the JAK/STAT pathway (GP130, JAK2, STAT3) remained transcriptionally unaltered. Our findings establish cardiac TCs as novel, intensity‐sensing cellular mediators of exercise‐induced physiological growth. The adaptive process, linked to stem cell activation, occurs without concomitant transcriptional upregulation of the core JAK/STAT signalling pathway components, suggesting the involvement of alternative, potentially non‐canonical, mechanistic pathways. This highlights the TC–cardiac stem cell axis as a potential target for optimizing exercise regimens for cardiac repair.

## Abstract (highlights)

- **What is the central question of this study?**
  Does exercise intensity differentially regulate cardiac telocytes (TCs) and is their expansion – potentially mediating physiological growth – dependent on the canonical JAK/STAT signalling pathway.
- **What is the main finding and its importance?**
  Exercise intensity proportionally expands cardiac TCs and activates stem cells, but without evidence of JAK/STAT pathway activation. This identifies TCs as novel intensity‐sensing cellular mediators and potentially redefines IL‐6's role, suggesting that alternative, non‐canonical pathways drive physiological growth.

## 1 INTRODUCTION

Exercise is a well‐established potent stimulus for physiological cardiac growth, promoting both cardiomyocyte hypertrophy and hyperplasia (Huang et al., 2025). While cardiac adaptation was historically attributed primarily to the enlargement of existing cardiomyocytes, emerging evidence demonstrates that exercise also activates cardiac stem cells (CSCs) and leads to *de novo* cardiomyogenesis (Leite et al., 2015; Marino et al., 2021; Waring et al., 2014). Despite these advancements, the precise cellular and molecular mechanisms by which exercise triggers CSC activation remain incompletely elucidated.

CSCs reside within specialized microenvironments known as niches, where their fate is meticulously regulated through intricate interactions with neighbouring cells (Pennings et al., 2018; Singh et al., 2019). Telocytes (TCs), a recently identified unique type of interstitial cell, have emerged as a crucial cellular component of these niches (Rosa et al., 2021). These cells, characterized by their extraordinarily long and thin extensions called telopodes (Cretoiu et al., 2019), form elaborate three‐dimensional networks throughout the heart (Tao et al., 2016). Through their telopodes, TCs establish intercellular connections and contribute to tissue homeostasis via paracrine secretion of cytokines, exosomes and microRNAs (Bei et al., 2016; Wei et al., 2022).

The strategic positioning of TCs within cardiogenic niches underscores their potential role as ‘nurse’ cells for CSCs. Indeed, growing evidence indicates that TCs support niche homeostasis and regulate CSC activity, proliferation and differentiation through their secretory activity (Bei et al., 2016). Recent investigations have demonstrated that cardiac TCs can secrete various cytokines, chemokines, and signalling molecules that modulate CSC behaviour (Condrat et al., 2021; Marini et al., 2017; Popescu & Fertig, 2016). Particularly relevant is evidence from co‐culture studies showing enhanced TC secretory activity when cultured with CSCs, with interleukin (IL)‐6 identified as a key mediator in this cellular communication (Albulescu et al., 2015). This finding suggests that cytokine secretion by TCs, including IL‐6, may represent an important mechanism for CSC activation.

The potential involvement of IL‐6 in TC‐mediated CSC activation warrants particular attention, as this myokine exhibits multiple functions in the cardiovascular system. Although traditionally considered an anti‐inflammatory molecule, IL‐6 can also induce hypertrophic effects on cardiomyocytes (Liu et al., 2023). Previous work has demonstrated IL‐6's involvement in stem cell‐mediated cardiac repair following myocardial injury (Mayfield et al., 2017). It appears that IL6 exerts these biological effects by binding to the GP130 receptor and activating downstream pathways (Scheller et al., 2011). One such signalling pathway is the Janus kinase (JAK)/signal transducer and activator of transcription (STAT) cascade, which has been implicated in cardiac hypertrophy through increased expression of STAT3 target genes (Pang et al., 2023).

Research in skeletal muscle physiology has established the JAK/STAT pathway's role in exercise‐induced hypertrophy (Begue et al., 2013; Ravalli et al., 2021; Trenerry et al., 2011), but its function in physiological cardiac growth remains ambiguous. Previous studies in healthy animal models have reported that exercise training increases IL‐6 and related cytokines while decreasing JAK2 and STAT3 expression (Chen et al., 2014), which suggests a complex regulatory mechanism for this pathway during cardiac adaptation. This paradoxical response highlights the need for further investigation into the JAK/STAT pathway's role in exercise‐induced cardiac growth.

Recent studies have begun to explore the response of cardiac TCs to exercise training, demonstrating that exercise training increases cardiac TC populations and that this increase correlates with CSC activation and physiological heart growth (Choobineh et al., 2023; Xiao et al., 2016). However, critical knowledge gaps remain. First, whether the TC response to exercise is intensity‐dependent remains unknown, despite intensity being a key parameter known to modulate cardiac adaptation (Wang et al., 2010; Waring et al., 2014; Wisløff et al., 2009). Second, the specific molecular mechanisms through which TCs influence CSCs in the context of exercise‐induced cardiac growth have not been systematically investigated. Therefore, this study was designed to investigate the intensity‐dependent response of cardiac TCs to interval training and to elucidate the potential involvement of the JAK/STAT pathway in TC‐mediated CSC activation.

## 2 METHODS

### 2.1 Ethical approval

All experimental procedures were approved by the Research Ethics Committee of Sport Sciences Research Institute of Iran (approval number: IR.SSRI.REC.1397.276) and conducted in accordance with the National Institutes of Health (NIH) *Guide for the Care and Use of Laboratory Animals*.

### 2.2 Animals and experimental design

Twenty‐four male Wistar rats (8 weeks old, mean body weight 232±33 g) were obtained from the Pasteur Institute of Iran. They were randomly assigned to three equal groups: control (CTRL, initially *n* = 8), high‐intensity interval training (HIIT, *n* = 8), and low‐intensity interval training (LIIT, *n* = 8). Rats were housed in polycarbonate cages under controlled conditions (12:12 h light–dark cycle, $22^{\circ }C$, 50±10% humidity) with free access to standard food and water ad libitum.  All animals were acclimatized to the laboratory environment for 1 week prior to initiation of the exercise protocol.

### 2.3 Exercise training protocols

The exercise protocols were designed based on established rodent training models (Hoydal et al., 2007; Kemi et al., 2005; Waring et al., 2014), with training intensity prescribed as percentages of maximal oxygen consumption (${\dot{V}}_{O_{2}\max}$). The exercise intensities were determined based on previously established velocity–${\dot{V}}_{O_{2}\max}$ relationships for Wistar rats (Hoydal et al., 2007). Over an 8‐week period, both exercise training groups completed five sessions per week on a motorized treadmill with 0° inclination. The HIIT protocol consisted of 60‐min sessions comprising a 5–10 min warm‐up at 50–60% ${\dot{V}}_{O_{2}\max}$, followed by five 8‐min high‐intensity intervals at 85–90% ${\dot{V}}_{O_{2}\max}$ interspersed with 2‐min active recovery periods at 50–60% ${\dot{V}}_{O_{2}\max}$, and concluding with a 5‐min cool‐down. Similarly, the LIIT protocol involved 60‐min sessions with a 5–10 min warm‐up at 45–50% ${\dot{V}}_{O_{2}\max}$, followed by five 8‐min intervals at 55–60% ${\dot{V}}_{O_{2}\max}$ separated by 2‐min active recovery periods at 45–50% ${\dot{V}}_{O_{2}\max}$, and ending with a 5‐min cool‐down period (Kemi et al., 2005; Waring et al., 2014). To ensure protocol adherence and animal welfare, all rats underwent a 1‐week familiarization period with the treadmill at low speeds prior to the main training. During the training sessions, if an animal slowed or stopped, mild tactile stimulation (gentle tapping on the hindquarters) was applied sparingly to encourage running. Predefined exclusion criteria were established for animals that consistently refused to run or exhibited clear signs of excessive stress (e.g., vocalization, freezing). No animals met these exclusion criteria, and all rats in the exercise groups successfully completed the 8‐week training protocol.

### 2.4 Tissue collection

Forty‐eight hours after the final training session, body weights were recorded, and animals were anaesthetized with an intraperitoneal injection of ketamine (90 mg/kg) and xylazine (5 mg/kg). The depth of anaesthesia was confirmed before any invasive procedure by the absence of both pedal withdrawal and corneal reflexes. A median sternotomy was performed under sterile conditions. While the animals were maintained under deep anaesthesia, the hearts were rapidly excised. Exsanguination and cardiac excision under deep anaesthesia served as the method of euthanasia. Total heart weight was immediately measured using a precision analytical balance. The heart‐to‐body weight ratio was calculated for each animal. Left ventricular tissue samples were either snap‐frozen in liquid nitrogen for molecular analyses (stored at −80°C) or fixed in 10% neutral buffered formalin for histological examination. One heart from the CTRL group was excluded from all subsequent analyses due to a technical failure during the initial tissue processing, which compromised sample integrity. Thus, the final group sizes for data analysis were *n* = 7 for CTRL and *n* = 8 for HIIT and LIIT.

### 2.5 Haematoxylin and eosin staining

Haematoxylin and eosin (HE) staining was performed to assess the left ventricular (LV) wall thickness, as well as the area and number of cardiomyocytes. Cardiac tissues were fixed in 10% neutral buffered formalin for 24 h at room temperature. Following fixation, tissues were dehydrated through a graded ethanol series (70%, 80%, 90%, 95% and 100%), cleared in xylene, and embedded in paraffin. Serial sections of 5 µm thickness were cut using a rotary microtome (Leica RM2235; Leica Biosystems, Nußloch, Germany) and mounted on glass slides. For HE staining, sections were deparaffinized in xylene and rehydrated through a descending ethanol series. Nuclei were stained with haematoxylin followed by eosin counterstaining for cytoplasmic visualization. After staining, sections were dehydrated, cleared in xylene, and mounted with a synthetic resin. Finally, the sections were examined and imaged under a standard bright‐field light microscope (Olympus BX53, Olympus, Tokyo, Japan).

Left ventricular wall thickness was measured at three equidistant points along the mid‐ventricular region using ImageJ software (v1.48, NIH, Bethesda, MD, USA). Morphometric analyses were performed using a systematic random sampling approach to ensure representative quantification. For cardiomyocyte cross‐sectional area measurement**, **three non‐consecutive sections from the mid‐ventricular region of each animal were analysed. From each section, ∼35 cardiomyocytes were randomly selected based on the following criteria: (1) circular or oval profile with intact cellular membrane; (2) visible central nucleus; and (3) clear cellular boundaries. In total, at least 100 cardiomyocytes per animal were analysed using ImageJ software. For cardiomyocyte numerical density assessment**, **10 non‐overlapping high‐power fields (×400 magnification) were systematically selected from each section by moving the microscope stage in a predetermined grid pattern, avoiding large vessels and fibrous areas. Only nuclei within clearly defined cardiomyocyte boundaries were counted. All morphometric analyses were performed by two independent investigators blinded to the experimental groups, and the average of their counts was used for statistical analysis.

### 2.6 Real‐time PCR analysis

The expression of IL‐6, cardiotrophin‐1 (CTF1), GP130, JAK2, STAT3 and GATA4 genes and ANP and BNP mRNA was examined using the real‐time PCR technique. In this method, total RNA was isolated from approximately 30 mg of LV tissue using Qiazol reagent (Qiagen, Hilden, Germany) according to the manufacturer's instructions. RNA concentration and purity were assessed spectrophotometrically, and samples with an *A*_260_/*A*_280_ ratio of ∼2.0 were used for subsequent analysis. Genomic DNA was removed using DNase I treatment (Thermo Fisher Scientific, Waltham, MA, USA). Subsequently, 1 µg of total RNA was reverse‐transcribed into first‐strand cDNA using the RevertAid First Strand cDNA Synthesis Kit (Thermo Fisher Scientific) with oligo (dT)18 primers.

Quantitative real‐time PCR (qPCR) was performed using SYBR Green PCR Master Mix (Thermo Fisher Scientific) on an ABI Step One Real‐Time PCR System (Thermo Fisher Scientific). Each 20 µL reaction contained 10 µL of 2× SYBR Green Master Mix, 0.5 µL of each forward and reverse primer (10 pmol/µL), 1 µL of cDNA template and 8 µL of nuclease‐free water. The thermal cycling protocol consisted of an initial denaturation at 94°C for 20 s, followed by 40 cycles of 94°C for 20 s, primer‐specific annealing at 58–60°C for 30 s, and extension at 72°C for 30 s. A melting curve analysis was conducted post‐amplification to confirm the specificity of the PCR products. The primer sequences for all target genes and the reference gene are listed in Table 1. Prior to the main experiment, the annealing temperature was optimized for each primer set, and 59°C was determined to be the optimal temperature for efficient amplification of all target genes. The amplification efficiency for each primer pair was validated to be between 90% and 110%. Gene expression levels were normalized to the housekeeping gene *GAPDH* and relative quantification was calculated using the $2^{-\Delta \Delta C_{t}}$ method. The CTRL group served as the calibrator for fold‐change calculations.

**TABLE 1:** Primer sequences used for quantitative real‐time PCR.

Gene symbol	Accession number	Primer sequence (5′ → 3′)	Product size (bp)
*Il6*	XM_017590638.1	F: GTCAGAGTGGGCAACAGAGAAG R: GACAAATAATGAGGTGGGCTGGG	201
*Ctf1*	NM_017129	F: ATG AAG TCT CCC GAG TCC AA R: TCA GCT CTG GGT TGG TTT CT	134
*Il6st* (GP130)	NM_001007667.1	F: AGCTTCCGGAAGAAGTACCG R: TGCCATCCACAACTGTCACT	118
*Jak2*	NM_031514.1	F: CAGATGGAGAGTATGTTGCCGA R: GTGGAAGAGATGATTGGGTGGA	154
*Stat3*	XM_006247257.3	F: ACCAACGACCTGCAGCAATA R: ACACTCCGAGGTCAGATCCA	200
*Gata4*	XM_017599789.1	F: TGTGCTAGAACTGGCAACCC R: CCTTGAGGGAGAAACAGCGT	214
*Nppa* (ANP)	NM_012612.2	F: CCTGGACTGGGGAAGTCAACC R: CTGGGCTCCAATCCTGTCAATCC	61
*Nppb* (BNP)	NM_001287348.2	F: CGGGGTGAGGTTGTTTTAGGA R: GTGGGAAGTTTGTGCTGGAAGA	79
*Gapdh*	XM_017593963.1	F: CATACTCAGCACCAGCATCACC R: AAGTTCAACGGCACAGTCAAGG	121

### 2.7 Enzyme‐linked immunosorbent assay analysis

Cardiac IL‐6 protein levels were quantified using a commercial rat IL‐6 enzyme‐linked immunosorbent assay (ELISA) kit (MyBioSource, San Diego, CA, USA, cat. no. MBS2021530) according to the manufacturer's instructions. Tissue homogenates were prepared from LV samples, and assays were performed in duplicate. The sensitivity of the assay was <0.5 pg/mL.

### 2.8 Immunofluorescence staining

Cardiac TCs and cardiomyocytes were identified using immunofluorescence staining for specific markers. TCs were detected by dual staining with CD34–platelet‐derived growth factor receptor (PDGFR)‐α and CD34–PDGFRβ markers, while cardiomyocytes were identified using antibody against α‐actin. LV tissues were fixed in 4% paraformaldehyde for 24 h at room temperature, dehydrated through a graded ethanol series, cleared in xylene, and embedded in paraffin. Serial sections of 5 µm thickness were cut using a rotary microtome.

Sections were deparaffinized in xylene and rehydrated through a descending ethanol series. Antigen retrieval was performed using 2 M HCl for 30 min, followed by neutralization with borate buffer for 5 min. After washing with phosphate‐buffered saline (PBS), sections were permeabilized with 0.3% Triton X‐100 in PBS for 30 min and blocked with 10% goat serum for 1 h at room temperature. Sections were incubated overnight at 4°C with the following primary antibodies: rabbit anti‐CD34 (1:100; Abcam, Cambridge, UK, ab81289), mouse anti‐PDGFRα (1:100; Abcam, ab61253), mouse anti‐PDGFRβ (1:100; Abcam, ab32570) and mouse anti‐α‐actin (1:100; Abcam, ab7817). After washing, sections were incubated for 1.5 h at 37°C with appropriate secondary antibodies: goat anti‐rabbit IgG‐FITC (1:150; Abcam, ab6717) for CD34 detection, and goat anti‐mouse IgG–fluorescein isothiocyanate (FITC) (1:150; Abcam, ab6785) for PDGFRα, PDGFRβ and α‐actin detection. Nuclei were counterstained with propidium iodide. Sections were examined using an Olympus fluorescence microscope. Image acquisition was performed using a wide‐field fluorescence microscope (Olympus BX53). Digital images were captured with a Canon Power Shot camera attached to the microscope, using a ×40 objective lens (final magnification ×400). CD34–PDGFRα and CD34–PDGFRβ double‐positive cells were quantified as TCs in 10 random fields per section. Cardiomyocytes were counted based on α‐actin‐positive cells with visible nuclei. All quantifications were performed by two independent investigators blinded to the experimental groups.

### 2.9 Statistics

Data are presented as means ± standard deviation (SD). All statistical analyses were performed using SPSS software (version 20; IBM Corp., Armonk, NY, USA). The normality of data distribution was confirmed using the Shapiro–Wilk test, and homogeneity of variances was verified with Levene's test. For comparisons among the three experimental groups (CTRL, LIIT and HIIT), one‐way analysis of variance (ANOVA) was employed. When ANOVA revealed significant main effects, *post hoc* comparisons were conducted using Tukey's honestly significant difference (HSD) test to identify specific group differences. The significance level was set at *P* ≤ 0.05 for all statistical tests.

## 3 RESULTS

### 3.1 Exercise‐induced cardiac growth and molecular changes

**FIGURE 1:**
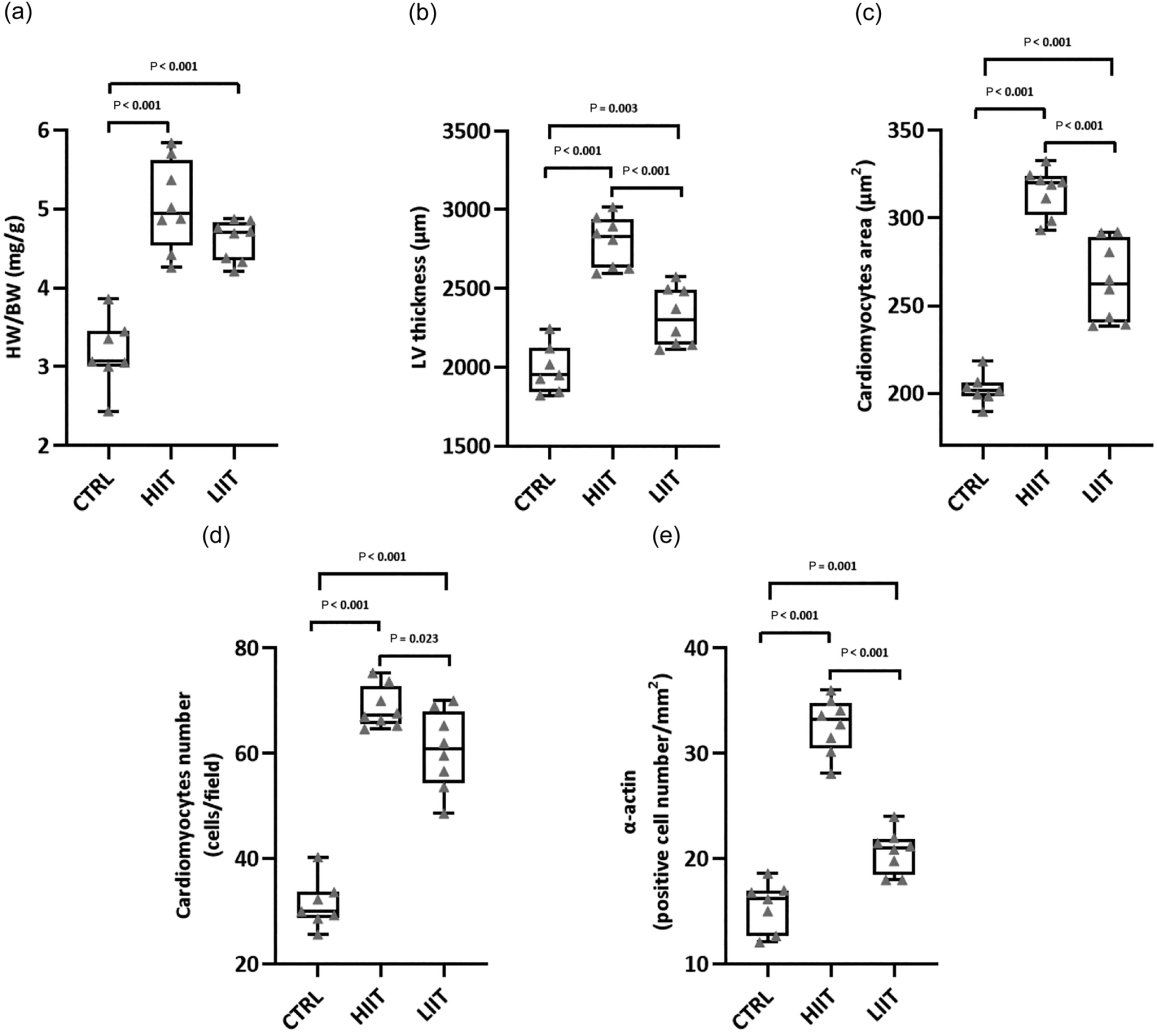
(a) Heart weight to body weight (HW/BW) ratio. (b) Left ventricular (LV) wall thickness. (c) Cardiomyocyte cross‐sectional area. (d) Cardiomyocyte numerical density. (e) Density of α‐actin‐positive cells. Data are box and whisker plots (min to max) with all individual data points shown (*n* = 7 for CTRL, *n* = 8 for HIIT and LIIT). The box shows the 25th–75th percentiles, the line is the median, and whiskers are min/max. Exact *P*‐values from Tukey's *post hoc* test are displayed on the graphs.

Exercise training significantly affected cardiac growth parameters (one‐way ANOVA). The heart weight‐to‐body weight (HW/BW) ratio showed significant differences among groups (*F*(2, 20) = 36.2, *P* < 0.001; *n* = 7 for CTRL, *n* = 8 for HIIT and LIIT). *Post hoc* analysis demonstrated that both HIIT (*P* < 0.001) and LIIT (*P* < 0.001) groups had significantly elevated HW/BW ratios compared to the CTRL group (Table 2, Figure 1a). Left ventricular wall thickness also exhibited significant intensity‐dependent augmentation (*F*(2, 20) = 45.1, *P* < 0.001; *n* = 7 for CTRL, *n* = 8 for HIIT and LIIT). Both HIIT (*P* < 0.001) and LIIT (*P* = 0.003) groups showed significant increases compared to the CTRL group, with the hypertrophic response to HIIT being significantly more pronounced than LIIT (*P* < 0.001; Figure 1b).

**TABLE 2:** Heart weight, body weight and heart weight to body weight ratio in experimental groups.

Group	Body weight (g)	Heart weight (g)	Heart weight/body weight (mg/g)
Control	320.5 ± 23.5	1.01 ± 0.11	3.17 ± 0.44
HIIT	288.5 ± 13.9	1.45 ± 0.11	5.04 ± 0.56
LIIT	304 ± 9.6	1.40 ± 0.11	4.60 ± 0.23

Data are presented as mean ± SD (*n* = 7 for CTRL, *n* = 8 for HIIT and LIIT).

**FIGURE 2:**
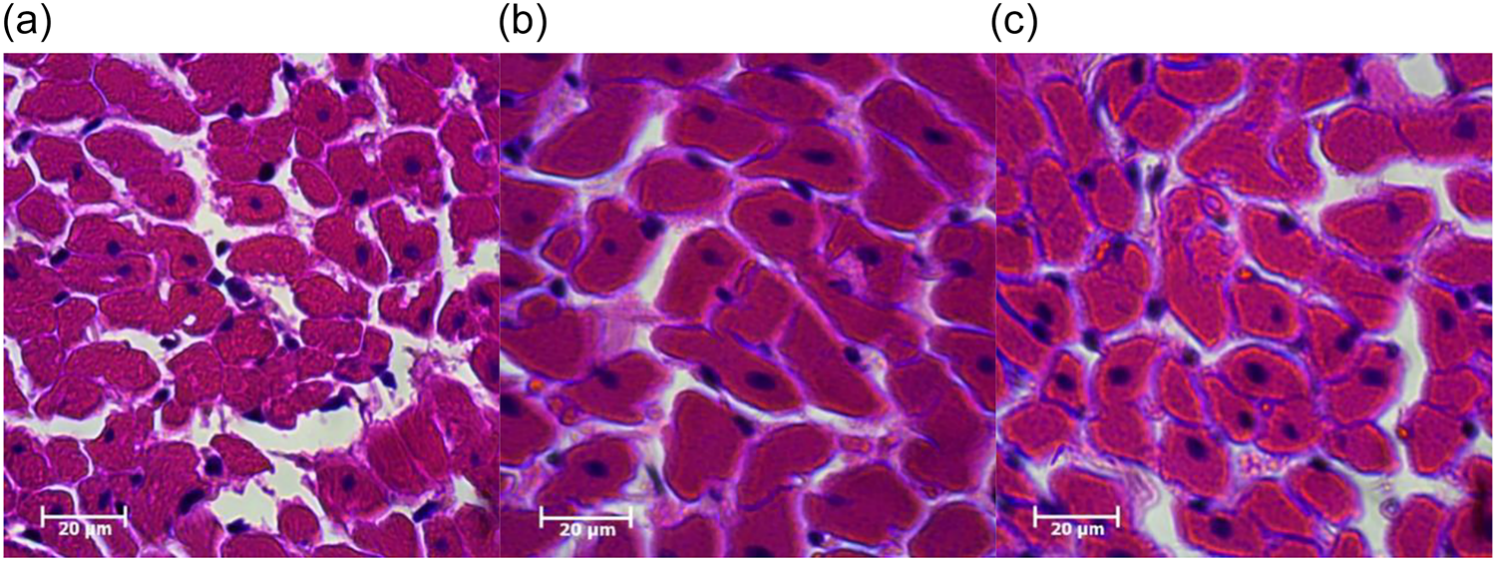
Exercise training increases cardiomyocyte size in an intensity‐dependent manner. Representative H&E‐stained sections of left ventricular cardiomyocytes from (a) CTRL, (b) HIIT, and (c) LIIT groups (scale bar: 20 µm). Quantitative analysis (Figure 1c) confirmed significant increases in cross‐sectional area in both exercise groups versus the CTRL group (*P* < 0.001), with HIIT inducing greater expansion than LIIT (*P* < 0.001). Images are representative of data from at least 100 cardiomyocytes per animal (*n* = 7 for CTRL, *n* = 8 for HIIT and LIIT).

**FIGURE 3:**
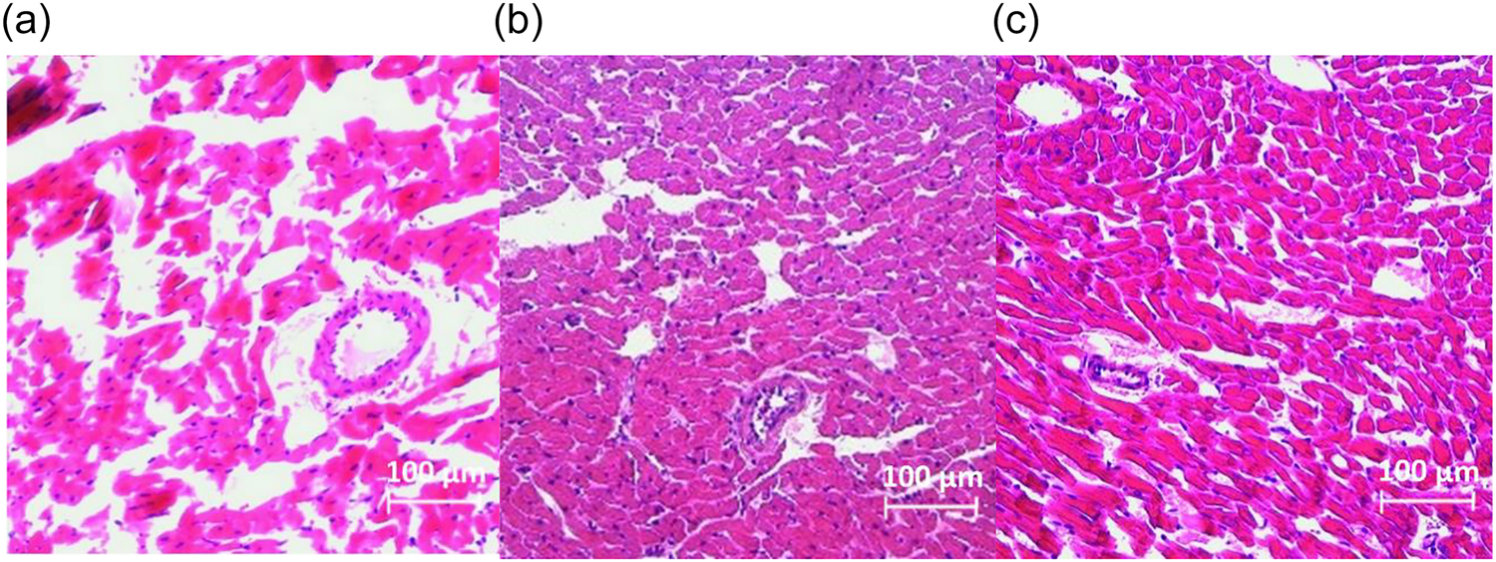
Exercise training increases cardiomyocyte number in an intensity‐dependent manner. Representative H&E‐stained sections of left ventricular cardiomyocytes from (a) CTRL, (b) HIIT, and (c) LIIT groups (scale bar: 100 µm). Quantitative analysis (Figure 1d) confirmed significant increases in cardiomyocyte number in both exercise groups versus CTRL (*P* < 0.001), with HIIT inducing greater cardiomyocyte number than LIIT (*P* = 0.023). Images are representative of data from at least 100 cardiomyocytes per animal (*n* = 7 for CTRL, *n* = 8 for HIIT and LIIT).

Cardiomyocyte size analysis revealed substantial increases in cross‐sectional area across groups (*F*(2, 20) = 90.5, *P* < 0.001; *n* = 7 for CTRL, *n* = 8 for HIIT and LIIT). Both exercise training groups showed significant enlargement compared to the CTRL group (both *P* < 0.001), with HIIT inducing greater cardiomyocyte enlargement than LIIT (*P* < 0.001; Figure 1c), with representative images shown in Figure 2. Total cardiomyocyte numbers were significantly elevated (*F*(2, 20) = 89.6, *P* < 0.001; *n* = 7 for CTRL, *n* = 8 for HIIT and LIIT) in both HIIT (*P* < 0.001) and LIIT (*P* < 0.001) groups compared to the CTRL group, with HIIT stimulating more substantial increases than LIIT (*P* = 0.023; Figure 1d), with representative images shown in Figure 3.

**FIGURE 4:**
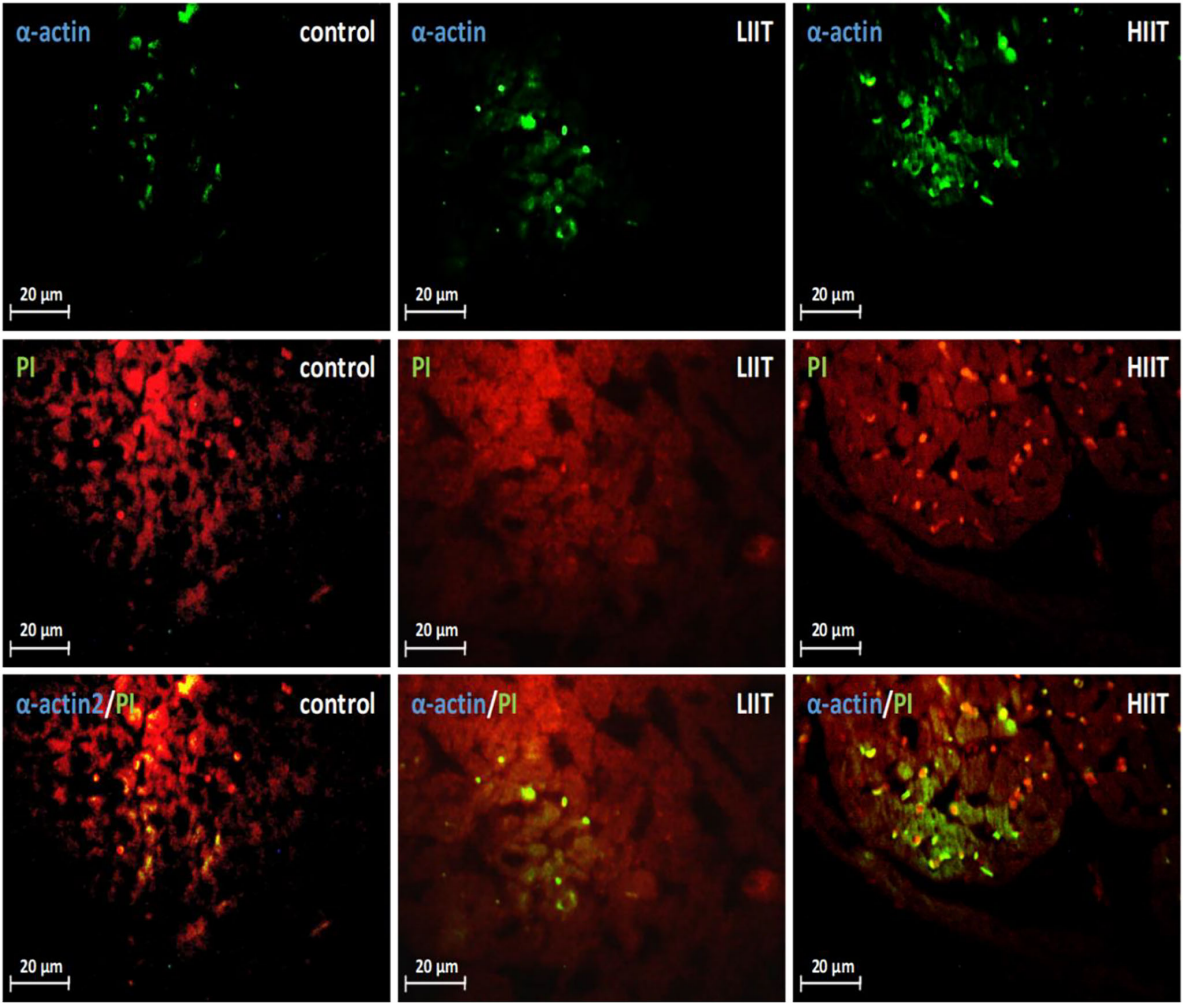
Changes in α‐actin‐positive cell density in response to exercise training. Representative immunofluorescence images showing α‐actin (green) and nuclei (propidium iodide (PI), red) in left ventricular sections. α‐Actin‐positive cells (cardiomyocytes) were quantified based on co‐localization with nuclei. Quantitative analysis (Figure 1e) confirmed significant increases in α‐actin‐positive cell density in both exercise groups versus CTRL (1.33‐fold, *P* = 0.001 and 2.11‐fold *P* < 0.001, respectively), with HIIT inducing greater α‐actin‐positive cell density than LIIT (*P* < 0.001).

Quantitative analysis of α‐actin‐positive cell density revealed a significant effect of exercise training (*F*(2, 20) = 107.23, *P* < 0.001; *n* = 7 for CTRL, *n* = 8 for HIIT and LIIT). *Post hoc* analysis indicated that both LIIT and HIIT induced a significant increase in α‐actin‐positive cell density compared to the CTRL group (1.33‐fold, *P* = 0.001 and 2.11‐fold *P* < 0.001, respectively). Furthermore, HIIT was significantly more effective than LIIT in increasing α‐actin‐positive cell density (*P* < 0.001; Figure 1e), with representative images shown in Figure 4.

**FIGURE 5:**
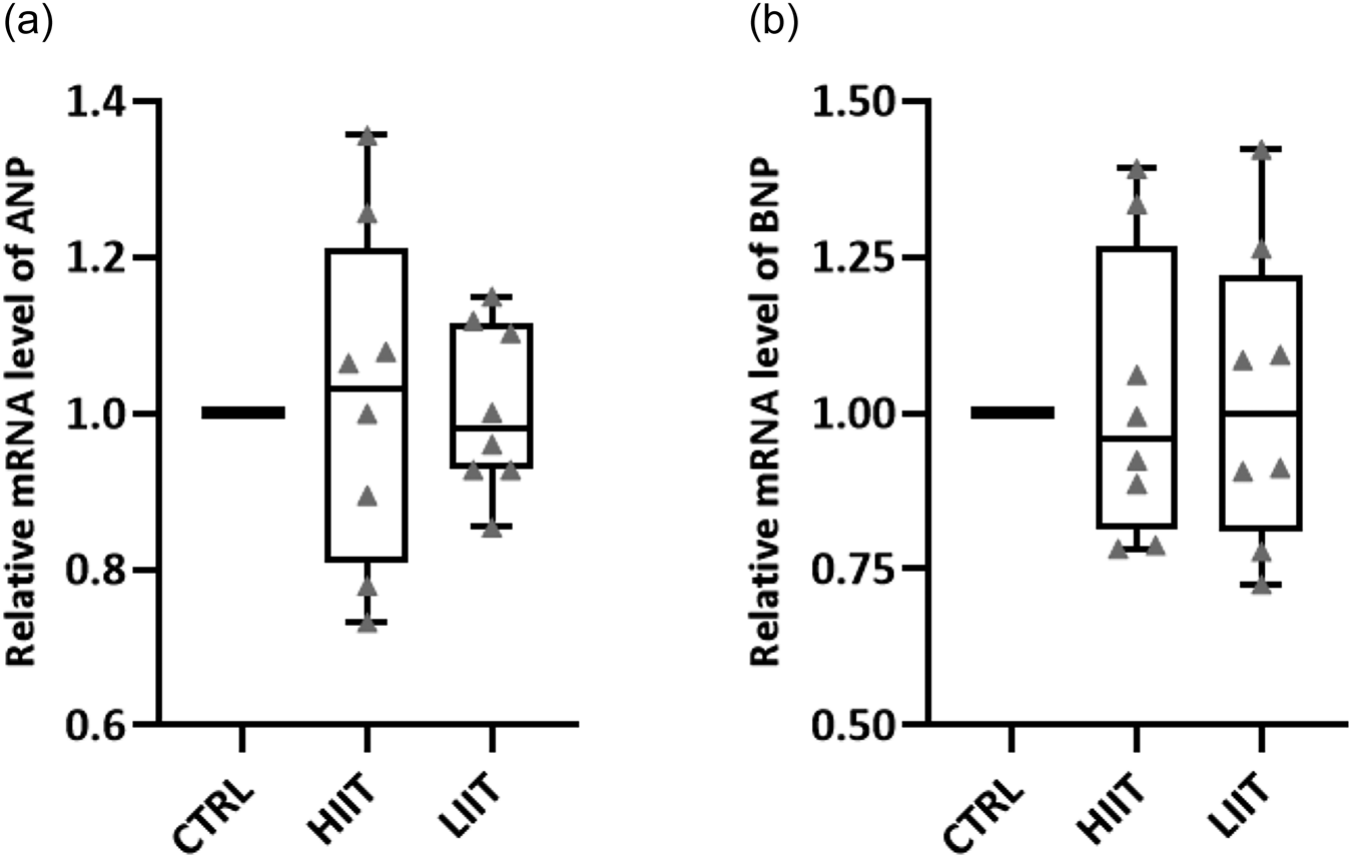
(a) Relative mRNA level of ANP. (b) Relative mRNA level of BNP. Data are presented as box and whisker plots (min to max) with individual data points overlaid (*n* = 7 for CTRL, *n* = 8 for HIIT and LIIT). The box represents the interquartile range (25th–75th percentile), the line indicates the median, and whiskers show the minimum and maximum values. No significant differences were observed among groups (ANP: *P* = 0.317; BNP: *P* = 0.086).

In contrast, pathological remodelling markers ANP and BNP mRNA levels remained unchanged following both training regimens (ANP: *F*(2, 20) = 1.21, *P* = 0.317; Figure 5a; *n* = 7 for CTRL, *n* = 8 for HIIT and LIIT and BNP: *F*(2, 20) = 2.78, *P* = 0.086; Figure 5b; *n* = 7 for CTRL, *n* = 8 for HIIT and LIIT), confirming the physiological nature of exercise‐induced cardiac growth.

### 3.2 Assessment of the JAK/STAT signalling pathway

**FIGURE 6:**
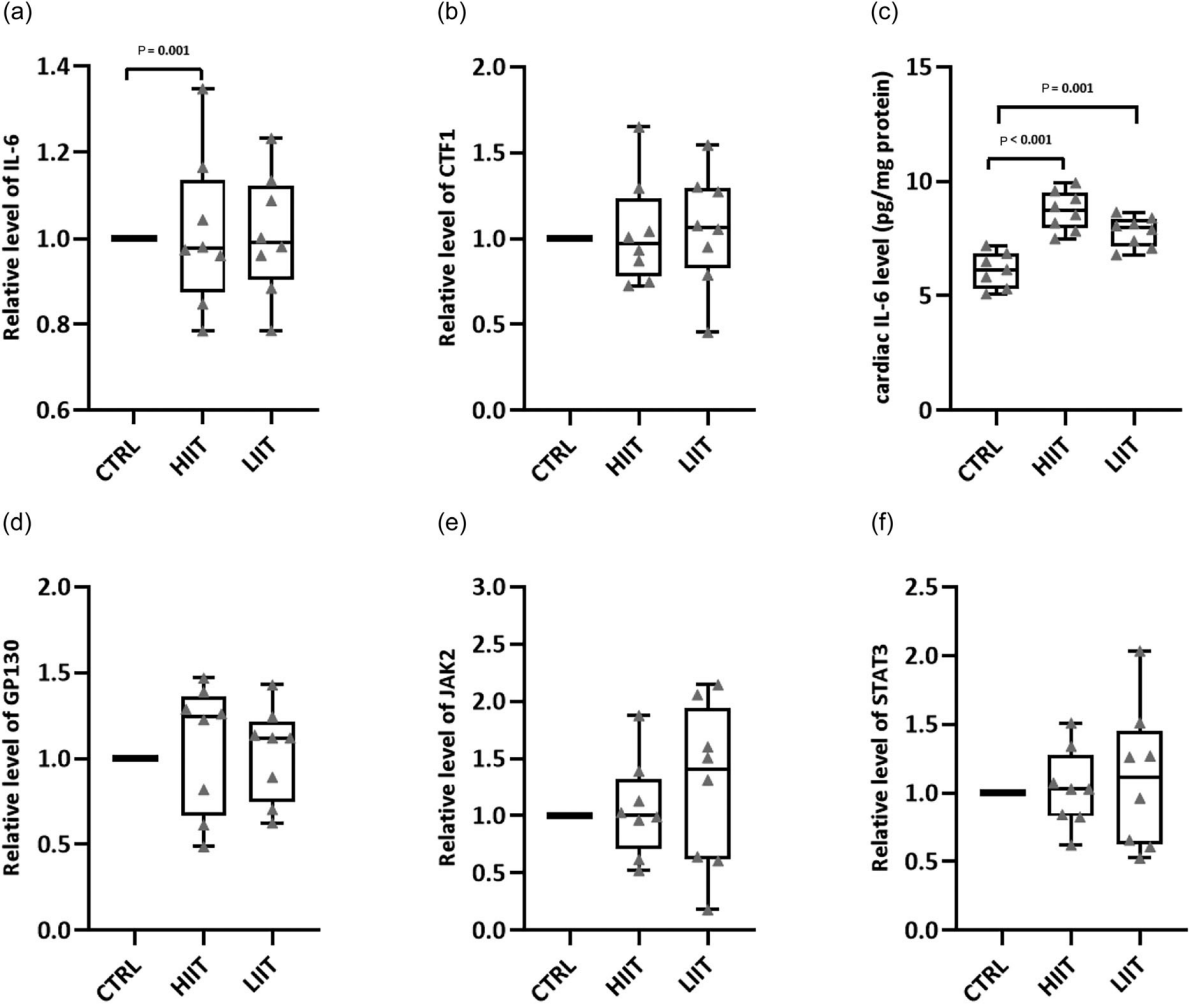
Relative changes in IL6 (a), CTF1(b), GP130 (d), JAK2 (e), and STAT3 (f) gene expression and cardiac IL‐6 protein level (c) changes in the HIIT and LIIT groups compared to the CTRL group after 8 weeks of exercise training in male rats. Data are presented as box and whisker plots (min to max) with individual data points overlaid (*n* = 7 for CTRL, *n* = 8 for HIIT and LIIT). The box represents the interquartile range (25th–75th percentile), the line indicates the median, and whiskers show the min and max values. Exact *P*‐values from Tukey's *post hoc* tests are shown for significant comparisons on the graphs. No significant differences were observed for CTF1 (*P* = 0.470), GP130 (*P* = 0.270), JAK2 (*P* = 0.839) or STAT3 (*P* = 0.366).

We next assessed the potential activation of the JAK/STAT pathway, a canonical downstream signalling cascade of IL‐6 family cytokines, in exercise‐induced cardiac growth. Analysis of the JAK/STAT pathway ligands revealed a distinct pattern. Cardiac IL‐6 gene expression showed significant differences among groups (*F*(2, 20) = 8.55, *P* = 0.002; *n* = 7 for CTRL, *n* = 8 for HIIT and LIIT), with significant upregulation in the HIIT group compared to the CTRL group (*P* = 0.001; Figure 6a). CTF1 gene expression was not significantly altered (*F*(2, 20) = 0.78, *P* = 0.470; *n* = 7 for CTRL, *n* = 8 for HIIT and LIIT; Figure 6b). Consistent with gene expression data, cardiac IL‐6 protein levels were significantly elevated (*F*(2, 20) = 21.66, *P* < 0.001; *n* = 7 for CTRL, *n* = 8 for HIIT and LIIT) in both HIIT (*P* < 0.001) and LIIT (*P* = 0.001) groups compared to CTRL (Figure 6c).

Despite these elevations in upstream ligands, the core components of the JAK/STAT pathway remained transcriptionally unaltered. No significant differences were observed in the gene expression of GP130 (*F*(2, 20) = 1.40, *P* = 0.270; *n* = 7 for CTRL, *n* = 8 for HIIT and LIIT; Figure 6d), JAK2 (*F*(2, 20) = 0.17, *P* = 0.839; *n* = 7 for CTRL, *n* = 8 for HIIT and LIIT; Figure 6e), or STAT3 (*F*(2, 20) = 1.05, *P* = 0.366; *n* = 7 for CTRL, *n* = 8 for HIIT and LIIT; Figure 6f) across experimental groups. This dissociation between increased ligand (IL‐6) availability and the lack of activation in its canonical signalling pathway provides no evidence for transcriptional activation of the canonical JAK/STAT cascade, suggesting it is unlikely to be the primary mediator under these conditions.

### 3.3 Exercise training increases cardiac TC population in an intensity‐dependent manner

**FIGURE 7:**
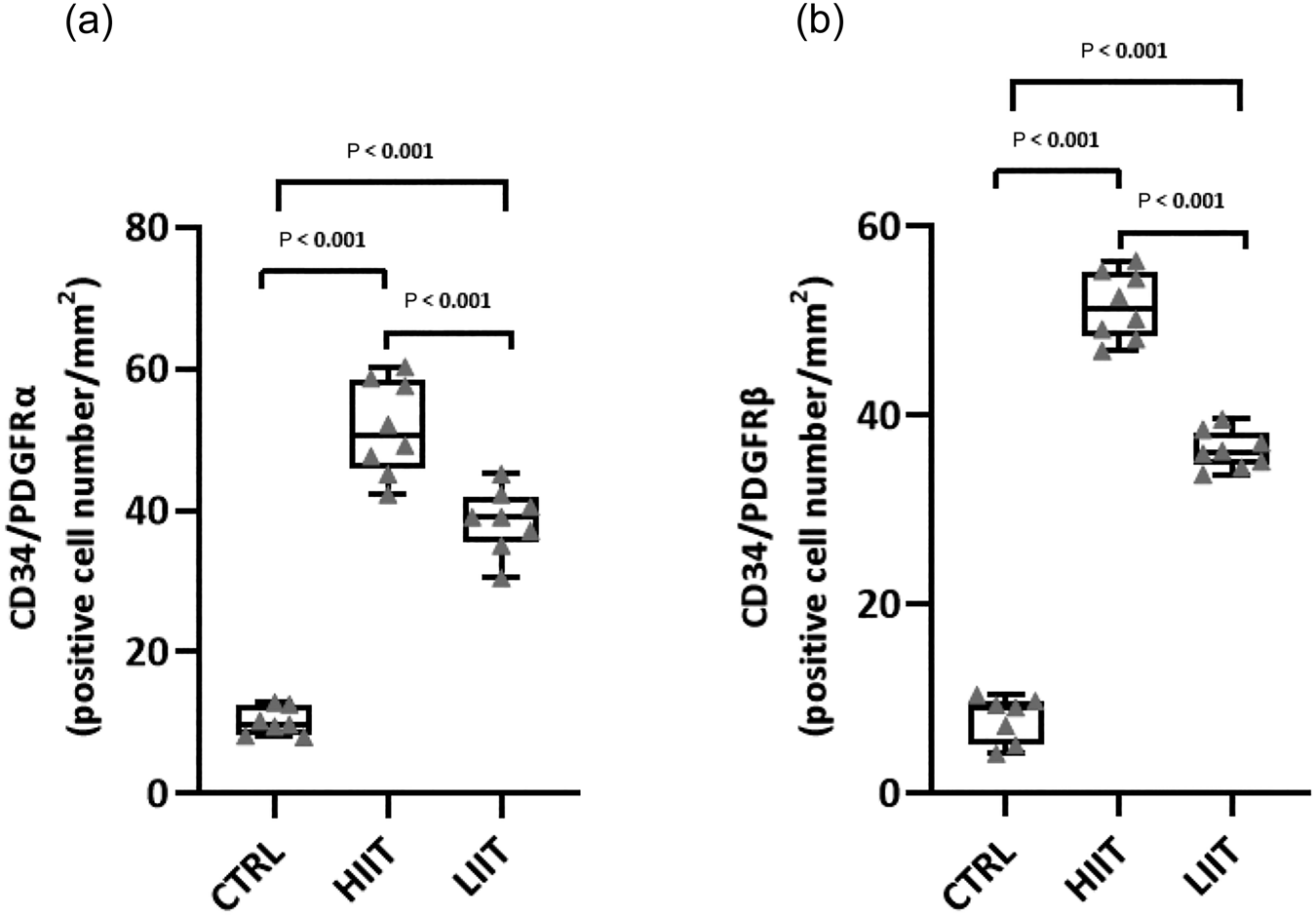
(a) Quantification of CD34–PDGFRα‐positive cells (TCs) per field. (b) Quantification of CD34–PDGFRβ‐positive cells (TCs) per field. Data are presented as box and whisker plots (min to max) with all individual data points shown (*n* = 7 for CTRL, *n* = 8 for HIIT and LIIT). The box shows the 25th–75th percentiles, the line is the median, and whiskers are min/max. Exact *P*‐values from Tukey's *post hoc* test are displayed on the graphs for significant comparisons.

**FIGURE 8:**
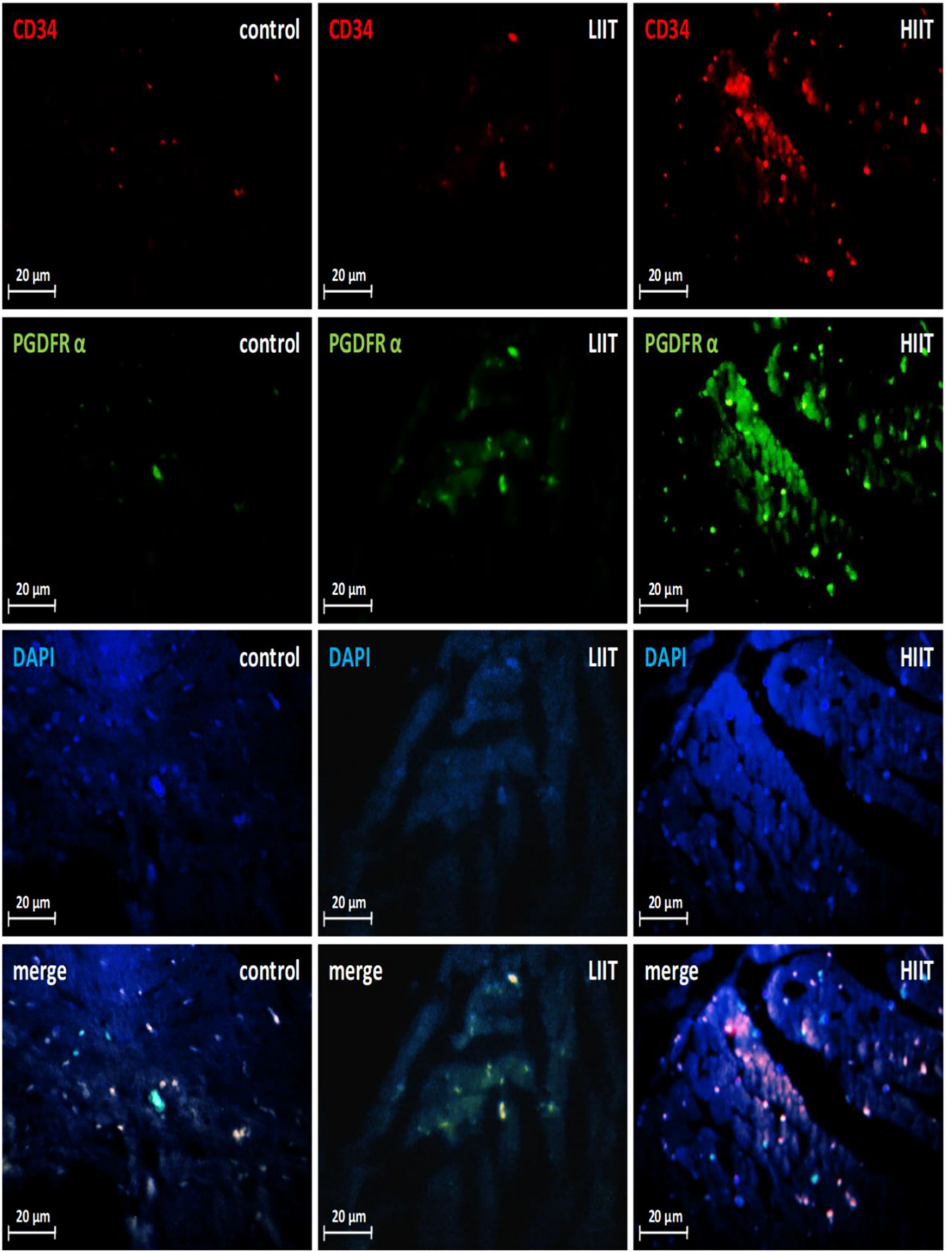
Changes in cardiac TCs in response to exercise training. CD34–PDGFRα‐dual staining images to determine cardiac TCs. Quantitative analysis (Figure 7a) confirmed significant increases in CD34–PDGFRα‐positive cells in both exercise training groups versus the CTRL group (*P* < 0.001), with HIIT showing greater increase than LIIT (*P* < 0.001). DAPI, 4′,6‐diamidino‐2‐phenylindole.

**FIGURE 9:**
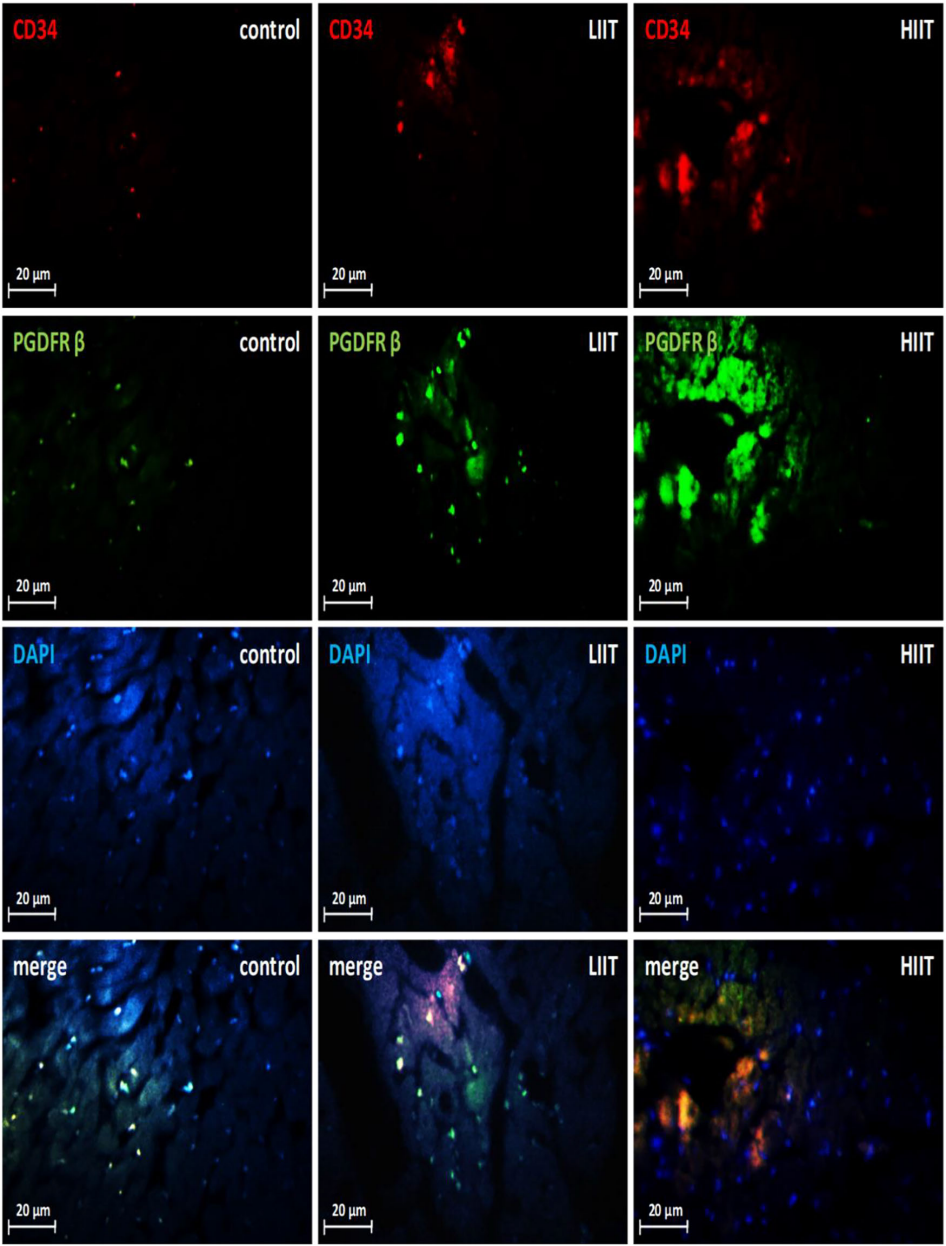
Changes in cardiac TCs in response to exercise training. CD34–PDGFRβ dual staining images to determine cardiac TCs. Quantitative analysis (Figure 7b) confirmed significant increases in CD34–PDGFRβ‐positive cells in both exercise training groups versus the CTRL group (*P* < 0.001), with HIIT showing greater increase than LIIT (*P* < 0.001). DAPI, 4′,6‐diamidino‐2‐phenylindole.

To investigate the potential role of TCs in exercise‐induced cardiac growth, we quantified their abundance in the left ventricle using immunofluorescence double‐staining for established TC markers (CD34–PDGFRα and CD34–PDGFRβ). Both exercise training protocols significantly expanded the cardiac TC population compared to the CTRL group for both CD34–PDGFRα‐positive (*F*(2, 20) = 139.36, *P* < 0.001; *n* = 7 for CTRL, *n* = 8 for HIIT and LIIT; Figures 7, 8) and CD34–PDGFRβ‐positive (*F*(2, 20) = 483.05, *P* < 0.001; *n* = 7 for CTRL, *n* = 8 for HIIT and LIIT; Figures 7, 9) cells. Notably, this exercise‐induced increase in TC abundance was intensity‐dependent. Quantitative analysis revealed that HIIT resulted in significantly greater numbers of both CD34–PDGFRα‐positive (*P* < 0.001) and CD34–PDGFRβ‐positive (*P* < 0.001) cells compared to the LIIT regimen.

### 3.4 Exercise intensity enhances GATA4 expression, a marker of cardiac stem cell activation

**FIGURE 10:**
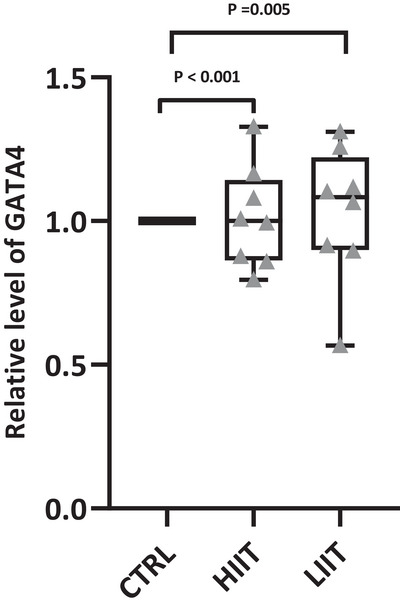
Relative changes in GATA4 gene expression changes in training groups compared to the CTRL group after 8 weeks of exercise training in male rats. Data are presented as box and whisker plots (min to max) with all individual data points shown (*n* = 7 for CTRL, *n* = 8 for HIIT and LIIT). The box shows the 25th–75th percentiles, the line is the median, and whiskers are min/max. Exact *P*‐values from Tukey's *post hoc* test are displayed on the graph for significant comparisons.

Consistent with the observed telocyte expansion and cardiomyogenesis, we found significant upregulation of GATA4, a key transcription factor in cardiac stem cell differentiation. GATA4 gene expression showed significant upregulation across groups (*F*(2, 20) = 14.41, *P* < 0.001; *n* = 7 for CTRL, *n* = 8 for HIIT and LIIT). Both exercise training groups exhibited elevated cardiac GATA4 expression compared to the CTRL group (HIIT: *P* < 0.001; LIIT: *P* = 0.005; Figure 10).

## 4 DISCUSSION

This study unveils a novel, intensity‐dependent axis governing physiological cardiac growth, centred on the expansion of cardiac TCs and their non‐canonical signalling to resident stem cells. We demonstrate that both high and low intensity interval training robustly promote physiological hypertrophy and cardiomyogenesis, with the magnitude of adaptation – including the proliferation of TCs and the upregulation of the stem cell differentiation marker GATA4 – being significantly greater following HIIT. Crucially, we present the first evidence that this TC‐mediated adaptive process operates independently of the JAK/STAT signalling pathway, despite a concurrent elevation in cardiac IL‐6, thereby challenging the canonical view of IL‐6's role in exercise‐induced cardiac remodelling and proposing a new mechanistic paradigm.

Our data firmly establish cardiac TCs as dynamic, intensity‐sensitive cellular components of the exercised heart. While previous studies noted TC expansion with exercise training (Choobineh et al., 2023; Ravalli et al., 2021; Xiao et al., 2016), the explicit dose–response relationship remained undefined. Here, we demonstrate that HIIT induces a significantly greater increase in CD34–PDGFRα/β‐positive TCs than LIIT. Basic studies have introduced TCs as dynamic sensors within the stem cell niche (Rosa et al., 2021). The findings of this study, by demonstrating an intensity‐dependent increase in the cardiac telocyte population, provide direct evidence that these cells actively sense exercise‐derived stimuli and their response is directly calibrated to exercise intensity. The parallel, intensity‐graded upregulation of GATA4, a master regulator of cardiogenesis (Almalki & Agrawal, 2016), provides compelling correlative evidence that the expanded TC network facilitates a more robust activation and differentiation of CSCs. This temporal coupling suggests TCs are not merely bystanders but active architects of a niche conducive to exercise‐induced cardiomyogenesis. This is consistent with the role of TCs as nurse cells within stem cell niches (Babadag & Çelebi‐Saltik, 2022), a function previously observed in cardiac regeneration contexts (Bei et al., 2016; Qin et al., 2024). Our findings extend the work of Xiao et al. (2016) and Choobineh et al. (2023), who reported increased cardiac TCs with exercise (Choobineh et al., 2023; Xiao et al., 2016), by quantitatively linking the magnitude of TC expansion to exercise intensity and its superior effect under HIIT.

We initially hypothesized the JAK/STAT pathway would be central to TC–CSC communication, given its established role in IL‐6 signalling and hypertrophy (Kumar et al., 2019; Pang et al., 2023). Our results, however, reveal a critical dissociation: robust increases in cardiac IL‐6 protein were accompanied by transcriptional quiescence in the core JAK/STAT machinery (GP130, JAK2, STAT3). This finding aligns with and extends the work of Chen et al. (2014), who observed JAK/STAT downregulation in healthy exercised rats, by demonstrating this pathway's inactivity within the context of a significantly expanded TC population. This suggests that the role of the JAK/STAT pathway may be context‐dependent, primarily engaged during pathological remodelling or disease states, as indicated by its activation in diseased models following exercise (Chen et al., 2014), but dispensable or suppressed during physiological growth in healthy myocardium. This finding appears to contrast with reports of JAK/STAT activation in skeletal muscle after exercise (Begue et al., 2013; Trenerry et al., 2011). This discrepancy underscores the tissue‐specific nature of exercise signalling. Moreover, beyond tissue differences, in pathological cardiac models where JAK/STAT is often hyperactivated, exercise can normalize its activity (Chen et al., 2014). Our data from healthy hearts suggest that in the context of pure physiological adaptation, the JAK/STAT pathway may not be requisite, pointing towards a fundamental difference in signalling topology between physiological and pathological growth.

This paradox necessitates a mechanistic reinterpretation. We propose that exercise‐induced IL‐6 – potentially secreted by TCs themselves (Albulescu et al., 2015) – signals through alternative, non‐canonical pathways to influence CSCs. Both IL‐6 and CT‐1 are potent activators of the mitogen‐activated protein kinase (MAPK) and phosphoinositide 3‐kinase (PI3K)/AKT pathways, which are pivotal regulators of cell survival, proliferation and hypertrophy (Fahmi et al., 2013; Rohini et al., 2010). We postulate that these pathways, rather than JAK/STAT, serve as the primary signalling conduits linking TC paracrine activity to CSC activation. Furthermore, TCs likely employ multi‐faceted communication strategies beyond cytokine secretion. As highlighted by Ravalli et al. (2021) in skeletal muscle, TCs contribute to tissue maintenance through intercellular signalling (Ravalli et al., 2021). Direct intercellular communication via telopodes and the targeted delivery of regulatory microRNAs and growth factors through exosomes (Babadag & Çelebi‐Saltik, 2022; Popescu et al., 2016) represent compelling alternative mechanisms that warrant future investigation. Our study shifts the focus from a single pathway to a network‐based model of TC‐mediated regulation.

Our discovery of an intensity‐dependent TC expansion is not an isolated phenomenon but rather a manifestation of a fundamental adaptive principle. This is powerfully corroborated by work in skeletal muscle, where Ravalli et al. (2021) demonstrated that TCs are a dynamic component of the tissue's response to use and disuse, with endurance training preventing their decline during inactivity (Ravalli et al., 2021). This parallel across tissues underscores TCs as a conserved regulatory component of the exercise‐adaptable cellular niche. Critically, the functional consequence of this expansion in the heart is likely the creation of a pro‐regenerative microenvironment. This is strongly supported by interventional studies in pathological models, where direct TC transplantation post‐myocardial infarction significantly enhanced cardiac recovery (Manole et al., 2011; Zhao et al., 2013). Our data, showing that this expansion is naturally inducible by exercise and coupled with increased GATA4 expression, provide a physiological and mechanistic bridge to these therapeutic observations. The expanded TC population is not just present; it is functionally active, fostering a niche that supports stem cell differentiation towards a cardiomyogenic fate.

This refined understanding, which positions the TC and the TC–CSC axis as a central therapeutic target for cardiovascular regeneration, carries immediate and profound clinical implications. Our data provide a robust physiological rationale for moving beyond the traditional debate on exercise modality. Instead, they spotlight exercise intensity as a key determinant of cellular niche adaptation. Harnessing this intensity–dose of TC expansion could inform novel rehabilitation strategies, advocating for the prescription of HIIT to maximize cardiac repair and health benefits in both preventive and therapeutic contexts.

While our findings provide a physiological rationale for optimizing exercise intensity to enhance cardiac regeneration, certain limitations should be considered. A key limitation of this interpretation is that it is based solely on gene expression data. We cannot rule out the possibility of transient changes in JAK/STAT protein levels or phosphorylation status that were not captured at our single post‐training time point. Furthermore, while our sample sizes (*n* = 7–8 per group) are consistent with comparable rodent exercise studies, the statistical power to detect subtle changes in gene expression may be limited. Future studies with larger cohorts would help confirm these observations. Future studies incorporating phospho‐protein analyses are essential to conclusively determine the activity status of this pathway in exercise‐induced physiological hypertrophy. Furthermore, the precise molecular cargo of TC‐derived exosomes and the specific receptors on CSCs that receive these signals remain to be elucidated. These limitations, however, do not detract from our central findings but rather chart a clear course for future research.

### 4.1 Conclusion

In conclusion, our study establishes cardiac TCs as exercise intensity‐sensing regulators of physiological cardiac growth. We demonstrate that exercise intensity proportionally expands the TC network, correlating with enhanced cardiac stem cell activation and cardiomyogenesis. Crucially, this TC‐mediated adaptation occurs in the absence of concomitant transcriptional upregulation in the core JAK/STAT components despite elevated IL‐6, pointing towards alternative mechanisms such as MAPK/PI3K pathways and vesicle‐mediated communication. However, the protein‐level activity status of this pathway requires further investigation to conclusively rule out its involvement. These findings provide a physiological basis for optimizing exercise intensity in preventive and regenerative strategies, while highlighting the need to define the precise molecular mediators of TC–CSC crosstalk.

## AUTHOR CONTRIBUTIONS

All authors contributed to study conception and design. approved the final version, and agree to be accountable for the work. All authors have read and approved the final version of this manuscript and agree to be accountable for all aspects of the work in ensuring that questions related to the accuracy or integrity of any part of the work are appropriately investigated and resolved. All persons designated as authors qualify for authorship, and all those who qualify for authorship are listed.

## CONFLICT OF INTEREST

None declared.

## FUNDING INFORMATION

None.

## Data Availability

The datasets generated during the current study can be obtained by contacting the corresponding author.
